# Analysis of the efficacy of sleeve gastrectomy, one-anastomosis gastric bypass, and single-anastomosis sleeve ileal bypass in the treatment of metabolic syndrome

**DOI:** 10.1038/s41598-024-54949-2

**Published:** 2024-03-01

**Authors:** Hang Yu, Lulu Qian, Yu Yan, Qi Yang, Xiaodong Shan, Youwei Chen, Xiao Fu, Xuehui Chu, Xing Kang, Xitai Sun

**Affiliations:** 1 Nanjing Drum Tower Hospital, Clinical College of Traditional Chinese and Western Medicine, Nanjing University of Chinese Medicine, Nanjing, 210008 China; 2 Department of Pancreatic and Metabolic Surgery, Nanjing Drum Tower Hospital, The Affiliated Hospital of Medical School, Nanjing University, Nanjing, 210008 China; 3 Department of General Surgery, Nanjing Drum Tower Hospital, Medical School of Southeast University, Nanjing, 210008 China; 4 Department of Rehabilitation and Dermatological Intervention, Nanjing Drum Tower Hospital, The Affiliated Hospital of Medical School, Nanjing University, Nanjing, 210008 China; 5 Southeastern University School of Medicine, Nanjing, 210003 China

**Keywords:** Endocrine system and metabolic diseases, Gastrointestinal diseases, Metabolic disorders

## Abstract

The objective of this study was to evaluate and compare the effectiveness of three different types of bariatric surgeries, namely, sleeve gastrectomy (SG), one-anastomotic gastric bypass (OAGB), and single anastomosis sleeve ileal (SASI) bypass, in the treatment of metabolic syndrome (MS). The optimal approach for managing MS remains uncertain, and thus this study aimed to provide a recent analysis of the efficacy of these surgical procedures. This retrospective study evaluated data of individuals who underwent SG, OAGB, and SASI bypass. The primary outcome measures included weight, body mass index (BMI), glucolipid metabolic index, and the occurrence of treatment-related complications within 6 to 12 months post-surgery. A total of 324 patients were included in this study. Of these, 264 patients underwent SG, 30 underwent OAGB, and 30 underwent SASI bypass. A significant decrease in weight was observed at the 6-month and 12-month marks following all three surgical procedures. Of these, patients who underwent SASI bypass exhibited the greatest reduction in weight and BMI post-surgery. Furthermore, the SASI bypass was associated with a significantly higher percentage of total weight loss (%TWL) and excess body mass index loss (%EBMIL) compared to SG and OAGB. Patients who underwent OAGB and SASI bypass demonstrated notable improvements in type 2 diabetes mellitus (T2DM). Patients who underwent SASI bypass and OAGB experienced greater postoperative comfort and reported fewer complaints of discomfort compared to the other procedure. Based on the retrospective analysis of the data, SASI bypass was associated with greater reductions in weight and BMI, higher percentages of %TWL and %EBMIL, and better improvement in T2DM compared to SG and OAGB. Therefore, both SASI bypass and OAGB were found to be more effective than SG in the treatment of MS.

## Introduction

Metabolic syndrome (MS) refers to a cluster of metabolic disorders characterized by disturbances in the metabolism of proteins, fats, carbohydrates, and other substances within the body. Its main manifestations include obesity, hypertension, diabetes, insulin resistance, and dyslipidemia. In recent years, great progress has been made in the treatment of MS, with bariatric surgery proving to be highly effective. This surgical approach not only leads to substantial weight reduction but also contributes to long-term survival, improved quality of life, and alleviation of obesity comorbidities such as type 2 diabetes, hypertension, and obstructive sleep apnea syndrome^1–3^. Sleeve Gastrectomy (SG), One Anastomosis Gastric Bypass (OAGB), and Single Anastomosis Sleeve Ileal Bypass (SASI) are commonly performed surgical procedures for the clinical management of MS^4–6^. In France and the United States, the advantages of SG in reducing complications have made it the procedure of choice for the treatment of patients with MS^7–9^. However, SG is also associated with many complications, such as gastric leakage, vomiting, and gastroesophageal reflux disease^10^. Furthermore, SG is associated with a higher incidence of weight loss failure^11^. OAGB is a modification of the conventional Roux-en-Y gastric bypass. Numerous studies have been conducted to assess the effectiveness and outcomes of OAGB^12,13^. The notable improvements in weight loss and management of comorbidities associated with it have resulted in its growing popularity among patients. SASI bypass is an emerging bariatric procedure^14^ that reduces anastomosis-related complications and shortens operative time by improving the technique of Sleeve Gastrectomy with Transient Bipartition (SG-TB) with a single anastomosis instead of using a Roux-en-Y anastomosis. This modification leads to a shorter duration of the surgical procedure. Nonetheless, its long-term data indicate a higher incidence of malnutrition. There is some controversy regarding the efficacy of the three surgical approaches. Therefore, the aim of this study is to compare the recent outcomes of the three surgical approaches in the treatment of patients with MS and serve as a reference for clinical treatment decisions in this regard.

## Methods

### Study design and patients

In this retrospective analysis, we examined the data of patients diagnosed with MS who underwent either SG, OAGB, or SASI bypass at our bariatric metabolic surgery department between January 2021 and January 2022. The patients were categorized into the following three groups based on the specific surgical modality: 264 cases in the SG group, 30 cases in the OAGB group, and 30 cases in the SASI bypass group. The preoperative evaluation included a review of medical history, physical examination, laboratory evaluation, and multidisciplinary consultation. All comorbidities such as type 2 diabetes, hypertension, hyperlipidemia, and sleep apnea syndrome were recorded in the database. Informed consent was obtained from all patients. Baseline characteristics, surgical outcomes, weight loss, and follow-up outcomes were included in the analysis. Regular follow-up visits were scheduled with the patients at specific intervals of 1 month, 3 months, 6 months, and 12 months during the first year. During these follow-up visits, laboratory assessments were performed to monitor weight loss progress and evaluate the nutritional status of the patients. All follow-up data from our center, as well as preoperative and perioperative data for each patient, were recorded in the database. The inclusion criteria for this study involved selecting individuals within the age range of 16 to 65 years with a body mass index (BMI) of ≥ 37.5 kg/m^2^ or ≥ 32.5 kg/m^2^ combined with one or more MS such as type 2 diabetes, obstructive sleep apnea syndrome, hyperlipidemia, hypertension, etc. Individuals who were unable to adhere to the prescribed strict post-operative diet or those who could not be regularly followed up as scheduled were excluded from the study. All patients included in the study were required to meet the criteria set by the Chinese Society for Metabolic and Bariatric Surgery (CSMBS) guidelines^15^ to undergo surgery. Patients signed an informed consent form related to surgery.

### Diagnostic criteria of MS

As per the MS diagnostic criteria published by the International Diabetes Federation (IDF) in 2005^16^, all included subjects met the following criteria: central obesity (waist circumference ≥ 90 cm in Chinese men and ≥ 80 cm in women) as a necessary condition, and any two of the following four factors: (1) triglyceride (TG) level ≥ 1.7 mmol/L or have been treated for this dyslipidemia; (2) reduced high-density lipoprotein cholesterol (HDL-c) level < 1.03 mmol/L in men and < 1.29 mmol/L in women or have been treated for this dyslipidemia; (3) elevated blood pressure: systolic blood pressure ≥ 130 mmHg or diastolic blood pressure ≥ 85 mmHg or have been diagnosed and started treatment for hypertension; and (4) fasting blood glucose (FPG) ≥ 5.6 mmol/L or have been diagnosed with type 2 diabetes.

### Surgical selection

Surgical selection for each patient was based on a shared decision between the patient and a multidisciplinary team that includes bariatric metabolic surgeons, endocrinologists, dietitians, psychologists, and anesthesiologists. When determining the appropriate surgical procedure for each patient, several factors were taken into consideration. These factors included the patient's baseline BMI, the presence of type 2 diabetes mellitus (T2DM), gastroesophageal reflux disease (GERD), eating behavior, and each patient's personal preferences. During the decision on the patient's surgical modality, patients were asked about their expectations and major concerns, and the advantages and disadvantages of each procedure were explained to the patient. For example, for younger patients (less than 30 years old), morbidly obese patients (BMI > 50 kg/m^2^), or patients with a combination of severe obstructive sleep apnea syndrome, we recommended the simpler and shorter SG procedure. On the contrary, our recommendation was to perform SASI bypass surgery for patients who exhibited a positive H. pylori infection or atrophic gastritis during endoscopy and had a family history of gastric cancer. Conversely, OAGB surgery was advised in other cases.

### Surgery

Patients were admitted to the hospital for further evaluation, including relevant imaging examinations such as chest X-rays, gastroscopy, and abdominal CT scans. Additionally, pertinent laboratory tests were conducted, including routine blood tests, liver and kidney function tests, coagulation function tests, and thyroid function tests. Following comprehensive communication with the patient, they were asked to sign an informed consent form for the surgery. Before the surgery, patients were instructed to fast for 8 h without consuming any food or water. Diabetic patients received insulin therapy to control their blood glucose levels.

### SG

**Figure 1:**
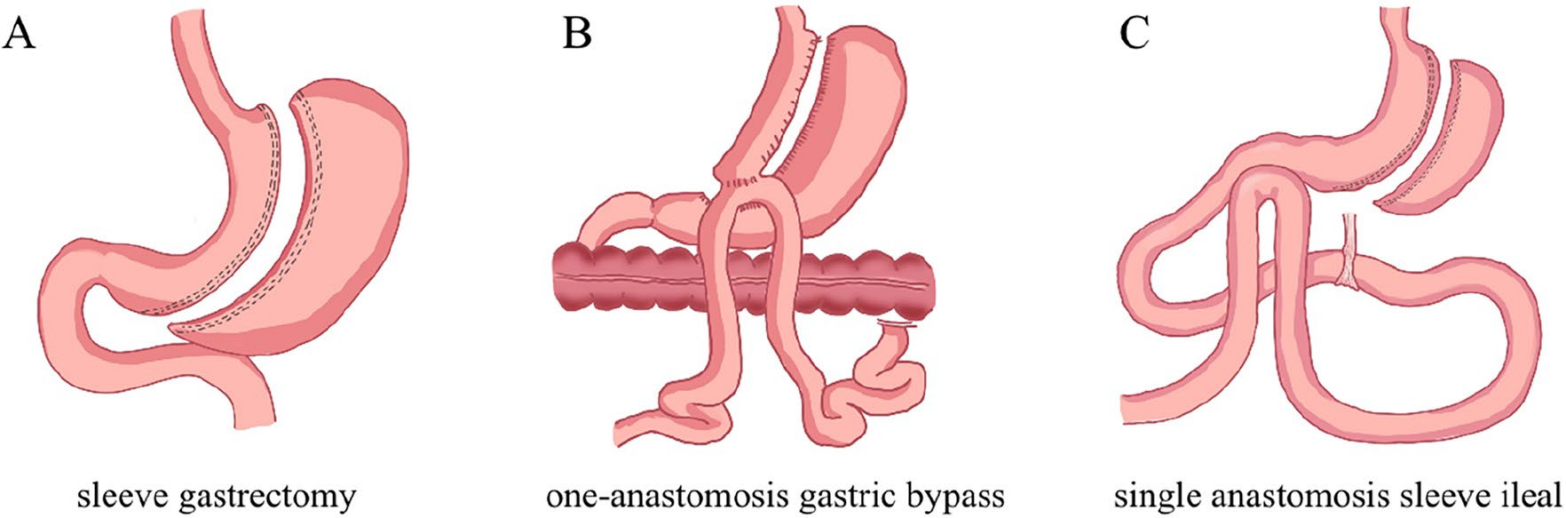
Schematic diagram of SG, OAGB, and SASI bypass [ (**A**) SG; (**B**) OAGB; (**C**) SASI bypass].

The patient was positioned in a supine position, and tracheal intubation was performed under general anesthesia. The patient's head was elevated, and the pneumoperitoneum was established using the 4-port technique method of operation. Using laparoscopic techniques, a dissection was performed approximately 4 cm from the pylorus. Branches of the right and left gastroepiploic vessels and short gastric vessels were divided along the lateral side of the greater curvature by carefully separating them from surrounding tissues. The fundus cardia and posterior wall of the stomach were completely liberated to expose the His angle. A 36Fr Bougie support tube was inserted, and the sleeve of the stomach was staple divided along the guiding tube in an upward direction. The resection of the gastric sleeve began at a point 4 cm from the pylorus, using the greater curvature side as the starting point. Up to the gastroesophageal junction, the fundus of the stomach was completely resected, leaving the cardia intact. To reinforce the gastric stump, continuous sutures were applied using 3-0 barbed sutures. Additionally, a laparoscopic drainage tube was inserted, and the incision sites were sutured (Fig. 1A).

### OAGB

The patient was positioned in a supine position with the head elevated and feet lowered. The 4-port technique was employed to create a pneumoperitoneum during the surgical procedure. Using laparoscopic visualization, the lesser omentum was opened on the lesser curve side of the stomach close to the gastric wall. The posterior gastric hiatus was separated immediately above the gastric wall to the left of the cardia. The Stapling was performed in a direction perpendicular to the long axis of the esophagus, starting from the less curved side of the separation, marking the completion of the initial cutting step. In the second step, a 90° rotation was made in the direction of the initial incision near the cardia, gradually separating the gastric wall. The jejunum was measured to a length of 200 cm from the Treitz ligament, where a lateral gastrojejunostomy was performed. This involved creating a 30 mm diameter anastomosis. The common opening resulting from the anastomosis was closed by manual suturing to strengthen both the stump and the anastomosis. The anastomosis was carefully inspected for any issues and to ensure adequate hemostasis. Subsequently, a laparoscopic drainage tube was inserted, and the incision sites were sutured (Fig. 1B).

### SASI bypass

The patient was positioned in a supine position with the head elevated and feet lowered. A sleeve gastrectomy procedure was performed approximately 7 cm from the pylorus. Around 2.6 m from the ileocecal region, the small intestine was elevated to the side of the greater curvature of the gastric pouch. A manual lateral anastomosis was then created. The stump and anastomosis were sutured. The anastomosis was carefully inspected for any issues and to ensure adequate hemostasis. Subsequently, a laparoscopic drainage tube was inserted, and the incision sites were sutured (Fig. 1C).

### Determination of efficacy

Based on the IDF criteria, we developed an index for determining the efficacy of MS after surgery.

### Statistics and analysis

SPSS 27.0 statistical software was used for data analysis and baseline comparisons were performed using Chi-square tests and independent sample t-tests. Continuous variables were expressed as means (standard deviations). Differences in patient characteristics were determined using the independent sample t-test.

### Ethical statement

This study was approved by the institutional review board of Nanjing Drum Tower Hospital Clinical College of Nanjing Medical University. All methods were carried out in accordance with the relevant guidelines and regulations in the manuscript.

## Results

### Patient characteristics

In this study, a cohort of 324 patients was included, and their baseline characteristics are summarized in Table 1. Among the patients, 224 (69.1%) were female, while 100 (30.9%) were male. The mean age, preoperative weight, and BMI of patients in the SG group were 30.05 ± 8.6 years, 109.7 ± 22.8 kg, and 38.7 ± 6.2 kg/m^2^, respectively; the mean age, preoperative weight, and BMI of patients in the OAGB group were 34.8 ± 5.6 years, 107.8 ± 20 kg, and 40.5 ± 4.5 kg/m^2^, respectively; the mean age, preoperative weight, and BMI of patients in the SASI group were 37.59 ± 8.8 years, 106.5 ± 19 kg, and 39.2 ± 5.1 kg/m^2^, respectively.

**Table 1:** Efficacy determination.

Efficacy Indicators	Complete relief	Partial relief	Invalid
Central obesity	Male < 90 cm Female < 80 cm	Less than preoperative value and Male ≥ 90 cm Female ≥ 80 cm	No change or increase in waist circumference compared to preoperative
Triglycerides (TG)	≤ 1.7 mmol/L and no lipid-lowering drugs	< Preoperative TG value and > 1.7 mmol/L, or reduce the dose of lipid-lowering drugs	≥ Preoperative TG value, or increase the dose and type of lipid-lowering drugs
High-density lipoprotein cholesterol (HDL-C)	Males < 1.0 mmol/L, females < 1.3 mmol/L and no lipid-lowering medication	< Preoperative HDL-C and ≥ 1.0 mmol/L for men and ≥ 1.3 mmol/L for women, or reduction in the dose and type of lipid-lowering medication	≥ Preoperative HDL-C, or increase the dose and type of lipid-lowering medication
Blood pressure	< 130/85 mmHg and no antihypertensive medication	< Preoperative blood pressure value and ≥ 130/85 mmHg, or reduction in the dose and type of antihypertensive medication	≥ Preoperative blood pressure value, or increase the dose and type of antihypertensive drugs
Fasting plasma glucose (FPG)	< 5.6 mmol/L and no glucose-lowering medication	< Preoperative FPG value and ≥ 5.6 mmol/L or reduced dose and type of glucose-lowering medication	≥ Preoperative FPG value, or increase the dose and type of glucose-lowering medication

The analysis of patient data revealed that the SG group had a younger age distribution, which can be attributed to the fact that sleeve gastrectomy is more frequently recommended for younger patients. In terms of comorbidities, the SG group had 108 (41.3%) cases of type 2 diabetes, while the OAGB and SASI bypass groups had 19 (63.3%) and 18 (62%) cases, respectively. This indicates that a higher proportion of patients with type 2 diabetes opted for OAGB and SASI bypass procedures. Furthermore, the OAGB group had a higher prevalence of hypertension, with 56.6% of patients in this group having hypertension compared to the other two groups (as indicated in Table 2).

**Table 2:** Demographics of patients in the three groups.

Variable	SG (*n* = 264)	OAGB (*n* = 30)	SASI bypass (*n* = 30)	*P*
Age	30.05 ± 8.6	34.8 ± 5.6	37.59 ± 8.8	0.103
Sex ratio (F:M)	189/75	22/8	13/17	0.202
BMI (kg/m^2^)	38.7 ± 6.2	40.5 ± 4.5	39.2 ± 5.1	0.214
Weight (kg)	109.7 ± 22.8	107.8 ± 20	106.5 ± 19	0.059
Waist (cm)	118 ± 12.9	115 ± 10.7	116.8 ± 11.2	0.187
Hips (cm)	120.8 ± 8.7	117.6 ± 8.3	119.7 ± 6.9	0.317
HbA1c (%)	6.1 ± 1.2	5.9 ± 1.1	6.9 ± 2.2	0.475
SBP (mmHg)	136.7 ± 18.3	138.9 ± 16.7	139.2 ± 12.8	0.102
DBP (mmHg)	84.2 ± 12.6	85.7 ± 11.8	85.2 ± 10.9	0.158
HDL-C (mmol/L)	1.4 ± 0.5	1.3 ± 0.8	1.4 ± 0.8	0.071
FPG (mmol/L)	6.01 ± 2.3	7.2 ± 3.2	7.1 ± 2.9	0.69
ALB (g/L)	42.5 ± 4.5	42.8 ± 2.4	42.5 ± 2.6	0.682
TG (mmol/L)	2.2 ± 2.5	2.0 ± 0.9	2.8 ± 1.8	0.301
Calcium (mmol/L)	2.4 ± 0.1	2.4 ± 0.1	2.4 ± 0.1	0.727
FINS (uIU/ml)	28.3 ± 14.4	20.7 ± 12.8	34.5 ± 17.9	0.986
C-PE (pmol/L)	1400.8 ± 516.8	1584.9 ± 475.7	1142 ± 473.5	0.073
Vit B12 (pg/ml)	483.5 ± 205.1	498.1 ± 234.9	643.3 ± 473.5	0.764
25 (OH)D	14.2 ± 6.3	18.1 ± 7.4	17.3 ± 5.3	0.982
Complications				
T2DM (%)	108 (41.3)	19 (63.3)	18 (62)	0.029*
Hypertension (%)	72 (27.5)	17 (56.6)	8 (20.7)	< 0.001*
Hyperlipidemia (%)	147 (56.1)	22 (73.3)	17 (58.6)	0.07
GERD (%)	29 (11.1)	3 (9.4)	3 (10.3)	0.859
OSAHS (%)	96 (36.4)	13 (43.3)	11 (36.7)	0.298

Data are presented as mean ± standard deviation.

*BMI* body mass index, *FPG* fasting plasma glucose, *ALB* albumin, *TG* triglycerides, *FINS* fasting insulin, *C-PE* fasting C-peptide, *SBP* systolic blood pressure, *DBP* diastolic blood pressure, *OSAHS* obstructive sleep apnea–hypopnea syndrome, *GERD* gastroesophageal reflux disease, *SG* sleeve gastrectomy, *OAGB* one-anastomosis gastric bypass, *SASI* single anastomosis sleeve ileal.

*p < 0.05.

### Perioperative discomfort complaints

The postoperative recovery of gastrointestinal-related functions, such as vomiting, acid reflux, bloating, and venting frequency, was assessed in all three groups. It was observed that the occurrence of vomiting was significantly lower in the SASI bypass group compared to the SG and OAGB groups. On the other hand, abdominal distension and acid reflux were more prevalent in the SG group compared to the OAGB and SASI bypass groups. Additionally, a higher number of patients in the OAGB group reported experiencing gastric colic compared to those in the SG and SASI groups (Table 3).

**Table 3:** Perioperative discomfort complaints in the three groups.

Variable	SG (n = 264)	OAGB (n = 30)	SASI bypass (n = 30)	*P*
Vomiting (%)	180 (68.2)	16 (53.3)	11 (36.7)	0.001*
Vent (times)	1.6 (0.6)	1.8 (0.4)	2.0 (0.5)	0.683
Acid reflux (%)	160 (60.6)	14 (46.7)	12 (40)	0.007*
Diarrhea (%)	13 (4.9)	2 (6.7)	7 (23.3)	0.002*
Gastric colic (%)	53 (20.1)	9 (30)	1 (3.3)	0.008*
Bloating (%)	153 (58)	2 (6.7)	1 (3.3)	0.008*

*p < 0.05.

### Weight loss

All three surgical procedures resulted in significant weight loss at the 6–12-month follow-up period, as evidenced by a significant decrease in weight and BMI compared to baseline values and a significant increase in %TWL and %EBMIL. Specifically, at the 12-month mark, patients who underwent SASI bypass showed a significant decrease in weight and BMI compared to those who underwent SG or OAGB procedures. Moreover, the SASI bypass was associated with a significantly higher percentage of total weight loss (%TWL) and excess body mass index loss (%EBMIL) compared to SG and OAGB, as indicated in Table 4.

**Table 4:** Weight loss observed at 6 months and 12 months postoperatively in the three groups.

Variable		SG (n = 264)	OAGB (n = 30)	SASI bypass (n = 30)	*P*
Weight (kg)	Preoperative	109.7 ± 22.8	107.8 ± 20	106.5 ± 19	0.059
	6 months postoperative	87.03 ± 17.06	93.09 ± 18.68	80.36 ± 15.87	0.028*
	12 months postoperative	79.8 ± 14.86	85.2 ± 10.35	70.1 ± 11.38	0.033*
	*P*	0.003*	< 0.001*	< 0.001*	
BMI (kg/m^2^)	Preoperative	38.7 ± 6.2	40.5 ± 4.5	39.2 ± 5.1	0.214
	6 months postoperative	30.86 ± 5.37	32.09 ± 3.71	28.4 ± 4.53	0.029*
	12 months postoperative	28.01 ± 4.39	29.87 ± 4.8	24.28 ± 3.79	0.022*
	*P*	0.002*	0.001*	< 0.001*	
%TWL	6 months postoperative	19.47 ± 3.72	21.51 ± 4.23	17.81 ± 5.2	0.006*
	12 months postoperative	26.47 ± 5.15	25.81 ± 3.91	27.73 ± 6.3	0.052
	*P*	< 0.001*	< 0.001*	0.004*	
%EBMIL	6 months postoperative	67.54 ± 36.08	56.62 ± 15	63.01 ± 37.13	0.024*
	12 months postoperative	84.09 ± 32.69	74.89 ± 27.9	88.23 ± 18.92	0.039*
	*p*	0.008*	< 0.001*	< 0.001*	

*%TWL* percentage of total weight loss, *%EBMIL* percentage of excess body mass index loss.

*p < 0.05.

### Changes in MS-related indicators

The postoperative efficacy of MS was determined based on the developed criteria. Significant changes in MS-related indicators were observed in all three groups during the 6- to 12-month postoperative period, as outlined in Table 5. In terms of central obesity, triglycerides, blood pressure, HDL cholesterol, and fasting glucose, all three groups showed obvious improvements after surgery. We also observed the insulin resistance index (HOMA-IR), an important index for assessing insulin sensitivity. Before surgery, the HOMA-IR values in the SG, OAGB, and SASI bypass groups were 7.28 ± 4.57, 5.46 ± 4.89, and 10.47 ± 6.72, respectively. Following surgery, these values decreased to 3.0 ± 0.8, 2.5 ± 0.7, and 2.6 ± 0.7, respectively, indicating varying degrees of improvement in insulin resistance among all three groups. The results of each of the above indicators were statistically significant (P < 0.05) in the postoperative groups compared with the preoperative ones.

**Table 5:** Changes in MS-related indicators.

Variable		SG (n = 264)	OAGB (n = 30)	SASI bypass (n = 30)	*P*
Waist (cm)	Preoperative	118 ± 12.9	115 ± 10.7	116.8 ± 11.2	0.187
	6 months postoperative	99.8 ± 11.3	100..8 ± 11.2	97.8 ± 9.2	0.023*
	12 months postoperative	97.6 ± 10.6	96.7 ± 10.9	95.3 ± 8.9	0.012*
	*p*	0.002*	0.001*	0.003*	
Hips (cm)	Preoperative	120.8 ± 8.7	117.6 ± 8.3	119.7 ± 6.9	0.317
	6 months postoperative	112.8 ± 7.2	113.3 ± 7.9	110.7 ± 9.4	0.037*
	12 months postoperative	108.3 ± 9.1	110.2 ± 8.7	105.8 ± 7.7	0.028*
	*p*	0.001*	0.003*	0.001*	
SBP (mmHg)	Preoperative	136.7 ± 18.3	138.9 ± 16.7	139.2 ± 12.8	0.102
	6 months postoperative	128.2 ± 10.4	125.3 ± 7.2	126.9 ± 6.1	0.087
	12 months postoperative	122.5 ± 5.8	120.4 ± 6.8	121.3 ± 7.2	0.079
	*p*	< 0.001*	0.002*	< 0.001*	
DBP (mmHg)	Preoperative	84.2 ± 12.6	85.7 ± 11.8	85.2 ± 10.9	0.158
	6 months postoperative	80.3 ± 6.8	81.7 ± 3.2	80.6 ± 5.9	0.214
	12 months postoperative	77.2 ± 3.9	77.1 ± 4.8	76.5 ± 3.4	0.207
	*p*	< 0.001*	< 0.001*	0.002*	
TG (mmol/L)	Preoperative	2.2 ± 1.5	2.0 ± 0.9	2.8 ± 1.2	0.301
	6 months postoperative	1.5 ± 0.4	1,6 ± 0.8	1.4 ± 0.2	0.012*
	12 months postoperative	1.3 ± 0.2	1.4 ± 0.4	1.2 ± 0.3	0.023*
	*p*	0.001*	0.002*	0.002*	
HDL-C (mmol/L)	Preoperative	1.4 ± 0.5	1.3 ± 0.8	1.4 ± 0.8	0.071
	6 months postoperative	1.2 ± 0.2	1.2 ± 0.6	1.2 ± 0.3	0..72
	12 months postoperative	1.1 ± 0.7	1.1 ± 0.8	1.1 ± 0.3	0.69
	*p*	0.031*	0.028*	0.019*	
FPG (mmol/L)	Preoperative	6.01 ± 2.3	7.2 ± 3.2	7.1 ± 2.9	0.69
	6 months postoperative	5.5 ± 1.8	5.0 ± 1.2	5.1 ± 1.3	0.002*
	12 months postoperative	5.0 ± 1.2	4.7 ± 0.8	4.8 ± 0.7	0.005*
	*p*	< 0.001*	< 0.001*	< 0.001*	
HOMA-IR	Preoperative	7.28 ± 4.57	5.46 ± 4.89	10.47 ± 6.72	0.71
	6 months postoperative	3.4 ± 1.6	3.8 ± 1.2	5.2 ± 0.8	0.012*
	12 months postoperative	3.0 ± 0.8	2.5 ± 0.7	2.6 ± 0.5	0.18
	*p*	< 0.001*	< 0.001*	< 0.001*	

*HOMA-IR* insulin resistance index.

*p < 0.05.

### Complete remission of MS-related complications

After a 12-month follow-up period, the treatment of MS and associated comorbidities demonstrated notable effectiveness, as evidenced by varying degrees of improvement in all the indicators across the three groups. In the SG group, 60 (22.7%) patients were identified as having a diagnosis of MS based on meeting all three diagnostic criteria, excluding those who had previously been diagnosed with MS. Among the 108 patients with preoperative concurrent type 2 diabetes mellitus, 65 (63.9%) experienced complete remission. Out of the 72 patients with preoperative concurrent hypertension, 54 (75%) achieved complete remission. Additionally, among the 147 patients with preoperative concurrent hyperlipidemia, 92 (62.6%) attained complete remission. Out of the 96 patients with preoperative concurrent sleep apnea syndrome, 90 (93.8%) patients achieved complete remission after the surgical intervention.

Following the exclusion of individuals already diagnosed with MS in the OAGB group, it was determined that 4 (13.3%) patients still met all three diagnostic criteria for MS. Among the 19 patients with preoperative concurrent type 2 diabetes, 18 (94.7%) patients achieved complete remission. Moreover, 16 (84.2%) patients had a significantly improved blood pressure profile. Among the 22 patients with preoperative combined hyperlipidemia, 17 (77.3%) patients achieved complete remission. The complete remission rate for sleep apnea syndrome also reached 92.3%.

After excluding individuals who met the three diagnostic criteria for MS in the SASI bypass group, only 2 patients (6.6%) remained diagnosed with MS. Complete remission was achieved in 83.3% of patients with type 2 diabetes, and complete remission was achieved in 87.5% of patients with hypertension. Complete remission was achieved in 13 out of 17 patients (76.5%) with preoperative hyperlipidemia, while the complete remission rate for sleep apnea syndrome in this group reached 100% (Table 6).

**Table 6:** Complete remission of MS-related comorbidities at 12 months postoperatively.

Variable	SG (n = 264)	OAGB (n = 30)	SASI bypass (n = 30)	*P*
T2DM (%)	65/108 (63.9)	18/19 (94.7)	15/18 (83.3)	0.03*
Hypertension (%)	54/72 (75)	16/17 (84.2)	7/8 (87.5)	0.001*
Hyperlipidemia (%)	92/147 (62.6)	17/22 (77.3)	13/17 (76.5)	0.002*
GERD (%)	16/29 (55.2)	3/3 (100)	3/3 (100)	0.009*
OSAHS (%)	90/96 (93.8)	12/13 (92.3)	11/11 (100)	0.91

*OSAHS* obstructive sleep apnea–hypopnea syndrome, *GERD* gastroesophageal reflux disease.

*p < 0.05.

### Change in biochemical parameters and nutritional status

In terms of nutritional status, serum albumin levels were significantly decreased after OAGB and showed a non-significant increase after SG and SASI bypass. However, the follow-up assessments showed that vitamin B12 and 25 hydroxyvitamin D levels were reduced in patients who underwent SASI bypass but remained within the normal range. In contrast, vitamin B12 and 25-hydroxyvitamin D remained within the normal range after SG and OAGB; therefore, the risk of postoperative malnutrition was higher in SASI compared with SG and OAGB (Table 7).

**Table 7:** Nutritional status at 12 months postoperatively.

Variable		SG (n = 264)	OAGB (n = 30)	SASI bypass (n = 30)	*P*
ALB (g/L)	Preoperative	42.5 ± 4.5	42.8 ± 2.4	42.5 ± 2.6	0.682
	12 months postoperative	44.89 ± 1.93	39.02 ± 12.44	44.97 ± 1.67	< 0.001*
	*p*	0.781	0.003*	0.158	
Calcium (mmol/L)	Preoperative	2.4 ± 0.1	2.4 ± 0.1	2.4 ± 0.1	0.727
	12 months postoperative	2.57 ± 0.15	2.7 ± 0.53	2.53 ± 0.06	0.035*
	*p*	0.791	0.028*	0.081	
Vit B12 (pg/mL)	Preoperative	483.5 ± 205.1	498.1 ± 234.9	643.3 ± 473.5	0.764
	12 months postoperative	549.01 ± 175.13	486.13 ± 111.69	574.43 ± 203.56	0.325
	*p*	0.319	0.587	0.06*	
25 (OH)D	Preoperative	14.2 ± 6.3	18.1 ± 7.4	17.3 ± 5.3	0.982
	12 months postoperative	22.35 ± 7.36	28.82 ± 14.04	15.64 ± 5.51	0.021*
	*p*	0.781	0.593	0.025*	

*25 (OH)D* 25-dihydroxy vitamin D.

*p < 0.05.

## Discussion

SG, OAGB, and SASI bypass are all effective bariatric procedures for weight loss. Nevertheless, it is important to note that these procedures employ different methods to achieve weight loss. SASI bypass, which is a relatively new procedure, deviates from the traditional bypass mechanism used in RYGB. Instead, it incorporates the concept of double bypass and involves a technical modification of the transabdominal bifurcated sleeve gastrectomy initially developed by Santoro et al. in 2012^17^.

All three surgical approaches have their advantages and disadvantages. Although SG is technically simpler to perform, it typically provides better outcomes in patients with a BMI < 50 kg/m^2^. However, there is a higher rate of weight regain after SG. Studies have shown that approximately 10% of patients may regain less than 5% of their initial weight at the 5-year mark following SG^18^. While OAGB demonstrated superior outcomes in terms of improving diabetes compared to SG, it was also associated with the occurrence of bile reflux and an increased risk of malnutrition^19^. In contrast, SASI bypass shows promising results in terms of weight loss and improvement of diabetes. However, since it is a relatively new procedure with limited studies available, it is currently considered an investigational approach and has not been fully established and evaluated. Further research is needed to assess its long-term effects on patients with MS.

While the majority of baseline characteristics among the patients were comparable across all three groups, there were still notable differences that could potentially influence the patients' ultimate selection of a surgical procedure. Patients who underwent SG were younger than those who underwent OAGB and SASI bypass. This age difference is significant because younger patients typically have a longer postoperative follow-up period. In the event of weight regain after surgery, younger patients have the advantage of being eligible for revision procedures to address and improve the condition. In contrast, patients with combined T2DM are more likely to choose OAGB, as it is primarily used as a metabolic procedure to improve T2DM. SASI bypass, being a combined procedure, is also favored by a significant number of patients with T2DM, and a recent study reported an improvement rate of more than 90% in T2DM after SASI bypass^14^.

In this study, we focused on the comfort and quality of life of patients after the three surgical procedures. Nausea and vomiting were reported by 54.8% of patients who underwent SG, and this can be attributed to the increased pressure in the remaining stomach and the narrowing of the gastric cavity caused by the SG procedure. Consistent with findings in the existing literature^20^, more than half of the patients in the SG group had significant postoperative bloating. Notably, our analysis revealed a higher prevalence of bloating among male patients compared to female patients. Further investigation is warranted to explore the underlying cause of this phenomenon. Regurgitation is the most common complication after SG^21^, and we recommend OAGB and SASI bypass for patients with combined GERD, but even so, severe regurgitation occurred in 68.4% of patients after SG. In contrast, patients who underwent SASI bypass experienced significant diarrhea, accompanied by an increase in the number of exhausts. Additionally, some patients reported an unpleasant odor associated with exhausts, which was considered unacceptable. Overall, the patients reported a higher level of comfort and better quality of life after undergoing OAGB. The main factor influencing patients' comfort was the occurrence of postoperative gastric colic, which we attribute to the potential excessive tension applied during the intraoperative gastrointestinal anastomosis. Additionally, we observed that male patients reported higher levels of postoperative comfort compared to female patients. Furthermore, we noted that older patients tended to have higher levels of comfort compared to younger patients. This difference in comfort levels may be attributed to a higher degree of tolerance among older patients. The current research on gender differences and age differences in relation to postoperative comfort after weight loss is limited, and there is a possibility of discovering new findings in this area through further investigations.

All three procedures resulted in significant weight loss, as indicated by progressive increases in %TWL and %EBMIL at 6 months of follow-up. The decrease in BMI and increase in %TWL at the 6-month mark after SG and SASI bypass were more significant compared to the outcomes observed with OAGB. According to Mahdy^22^, the ideal weight loss procedure should induce weight loss by implementing functional restriction and neuroendocrine control, which regulate hunger and satiety. This approach, as seen in SASI bypass, is preferable over procedures that rely solely on mechanical restriction and malabsorption, sub-defined as a digestive adaptation technique by Santoro as well^23^. Regarding the superior effect of SASI bypass on weight loss, it has been suggested that it is not only due to the restriction of gastric volume or the reduction of nutrient absorption, but more importantly, due to the neuroendocrine response generated by the early receipt of nutrients in the distal intestine, stimulating the secretion of hormones that produce satiety in the distal intestine, reducing proximal intestinal activity, and inducing hypothalamic-mediated satiety^24^. As of now, there is a lack of studies examining hormonal changes after SASI bypass. Further investigation through randomized controlled trials and prospective studies is necessary to explore this phenomenon in more detail.

In the SG group, a total of five patients were readmitted due to short-term postoperative complications. One patient experienced postoperative anemia, another patient had postoperative blood in the stool, and the remaining three patients were successfully discharged after receiving symptomatic treatment for postoperative gastrointestinal disorders. Only one patient in the OAGB group presented with vomiting blood. At the 6-month postoperative assessment, no instances of SASI bypass were observed, although there were some complications. These complications may be attributed to the duration of follow-up and the number of patients included in the study. In contrast, serious complications such as gastric leak^25^, bleeding^26^, anastomotic stricture^27^, and anastomotic ulcer^28^, were not observed in our study. We believe that the safety of bariatric surgery is primarily influenced by the following factors: a thorough preoperative multidisciplinary assessment, an intraoperative meticulous operation performed by skilled surgeons, postoperative professional care, and post-discharge follow-up by the case manager. At our center, we prioritize these aspects and take pride in having experienced lead surgeons, which significantly contributes to reducing the occurrence of postoperative complications.

This study has certain limitations including its retrospective design, lack of randomization, and focus on a single center. Although the lack of randomization resulted in differences in baseline characteristics among the three procedures being compared, the primary objective of this study was to develop customized weight loss surgery protocols tailored to the specific requirements of each patient. The results of this study represent the short-term outcomes of SG, OAGB, and SASI bypass in the treatment of MS. However, to fully evaluate the outcomes of the study and assess the long-term complications associated with each procedure, it is crucial to conduct further follow-ups over an extended period of time.

## Data Availability

The datasets produced and/or analyzed in the current study are not publicly accessible as they are stored within a database specific to a single center. However, interested individuals may request access to the data from the corresponding author, and reasonable requests will be considered.
