# Author Correction: Analysis of the efficacy of sleeve gastrectomy, one-anastomosis gastric bypass, and single-anastomosis sleeve ileal bypass in the treatment of metabolic syndrome

**DOI:** 10.1038/s41598-024-65287-8

**Published:** 2024-06-26

**Authors:** Hang Yu, Lulu Qian, Yu Yan, Qi Yang, Xiaodong Shan, Youwei Chen, Xiao Fu, Xuehui Chu, Xing Kang, Xitai Sun

**Affiliations:** 1 Nanjing Drum Tower Hospital, Clinical College of Traditional Chinese and Western Medicine, Nanjing University of Chinese Medicine, Nanjing, 210008 China; 2 Department of Pancreatic and Metabolic Surgery, Nanjing Drum Tower Hospital, The Affiliated Hospital of Medical School, Nanjing University, Nanjing, 210008 China; 3 Department of General Surgery, Nanjing Drum Tower Hospital, Medical School of Southeast University, Nanjing, 210008 China; 4 Department of Rehabilitation and Dermatological Intervention, Nanjing Drum Tower Hospital, The Affiliated Hospital of Medical School, Nanjing University, Nanjing, 210008 China; 5 Southeastern University School of Medicine, Nanjing, 210003 China

Correction to: *Scientific Reports* 10.1038/s41598-024-54949-2, published online 01 March 2024

The original version of this Article contained an error in Affiliation 1, which was incorrectly given as ‘Department of General Surgery, Taikang Xianlin Drum Tower Hospital, Affiliated Hospital of Medical School, Nanjing University, Nanjing, 210046, China’. The correct affiliation is listed below:

Nanjing Drum Tower Hospital, Clinical College of Traditional Chinese and Western Medicine, Nanjing University of Chinese Medicine, Nanjing, 210008, China

The original Article has been corrected.

